# Alkaline organosolv pretreatment of different sorghum stem parts for enhancing the total reducing sugar yields and *p*-coumaric acid release

**DOI:** 10.1186/s13068-020-01746-4

**Published:** 2020-06-10

**Authors:** Dandan Li, Liangkun Long, Shaojun Ding

**Affiliations:** The Co-innovation Center of Efficient Processing and Utilization of Forest Resources, Jiangsu Key Lab for the Chemistry & Utilization of Agricultural and Forest Biomass, College of Chemical Engineering, Nanjing Forestry University, Nanjing, China

**Keywords:** Sorghum stem, NaOH–ethanol pretreatment, *p*-Coumaric acid, Enzymatic hydrolysis

## Abstract

**Background:**

The sorghum stem can be divided into the pith and rind parts with obvious differences in cell type and chemical composition, thus arising the different recalcitrance to enzyme hydrolysis and demand for different pretreatment conditions. The introduction of organic solvents in the pretreatment can reduce over-degradation of cellulose and hemicellulose, but significance of organic solvent addition in pretreatment of different parts of sorghum stem is still unclear. Valorization of each component is critical for economy of sorghum biorefinery. Therefore, in this study, NaOH–ethanol pretreatment condition for different parts of the sorghum stem was optimized to maximize *p*-coumaric acid release and total reducing sugar recovery.

**Result:**

Ethanol addition improved *p*-coumaric acid release and delignification efficiency, but significantly reduced hemicellulose deconstruction in NaOH–ethanol pretreatment. Optimization using the response surface methodology revealed that the pith, rind and whole stem require different NaOH–ethanol pretreatment conditions for maximal *p*-coumaric acid release and xylan preservation. By respective optimal NaOH–ethanol pretreatment, the *p*-coumaric acid release yields reached 94.07%, 97.24% and 95.05% from pith, rind and whole stem, which increased by 8.16%, 8.38% and 8.39% compared to those of NaOH-pretreated samples. The xylan recoveries of pith, rind and whole stem reached 76.80%, 88.46% and 85.01%, respectively, which increased by 47.75%, 15.11% and 35.97% compared to NaOH pretreatment. Adding xylanase significantly enhanced the enzymatic saccharification of pretreated residues. The total reducing sugar yields after respective optimal NaOH–ethanol pretreatment and enzymatic hydrolysis reached 84.06%, 82.29% and 84.09% for pith, rind and whole stem, respectively, which increased by 29.56%, 23.67% and 25.56% compared to those of NaOH-pretreated samples. Considering the separation cost of the different stem parts, whole sorghum stem can be directly used as feedstock in industrial biorefinery.

**Conclusion:**

These results indicated that NaOH–ethanol is effective for the efficient fractionation and pretreatment of sorghum biomass. This work will help to understand the differences of different parts of sorghum stem under NaOH–ethanol pretreatment, thereby improving the full-component utilization of sorghum stem.

## Background

Lignocellulosic biomass is an important feedstock for the production of biofuels and biochemicals [1]. Cellulose, hemicellulose and lignin are the main biochemical components of lignocellulose. These three components are strongly intermeshing and bonded together (covalently or non-covalently) to form lignocellulosic matrix. Structural features make lignocellulosic biomass difficult to be deconstructed and digested, and result in relatively low utilization of lignocellulosic feedstock [2]. To improve the enzymatic digestibility of lignocellulosic biomass, various pretreatment methods have been investigated based on the properties of each lignocellulosic feedstock [3]. Common pretreatment methods include acid treatment, alkali treatment, organosolv treatment, automated hydrolysis treatment, steam explosion treatment, etc. [4]. Among these pretreatment methods, alkali pretreatment is widely used due to the advantages of non-corrosive, non-toxic, and efficient delignification [5, 6]. NaOH pretreatment is one of leading technologies for pilot-scale biorefinery of herbaceous lignocellulose [7]. However, in traditional NaOH pretreatment, half or even more amount of the hemicellulose is also dissolved in black liquor [8, 9].

In general, the purpose of the pretreatment is to remove lignin and hemicellulose, thereby increasing the enzymatic hydrolysis efficiency of cellulose [10]. Xylan is the main hemicellulose component occupying 20–30% of biomass of the hardwoods (dicots) and herbaceous plants [11]. Effective utilization of xylan can improve the rational utilization of sugars in lignocellulosic biomass, which leads to the production of high-yield fermentable sugar/ethanol [12, 13]. However, xylan is susceptible to alkali pretreatment processes and easier to be degraded than cellulose due to its amorphous and branched structure [14]. The dissolved xylan in the liquid streams was partially destroyed and it was difficult to be fully recovered and reutilized [15], therefore reducing the total sugar utilization. The use of organic solvents (such as ethanol) instead of water during alkaline pretreatment avoids the over-deconstruction of cellulose and hemicellulose, as well as the formation of conventional inhibitors [16].

Sorghum is one of the main bioenergy crops, and the biorefinery of its soluble sugar and bagasse residue for biofuels and biochemicals has attracted much attention in recent years [17–20]. Beside soluble sugar and lignocellulose residue, sorghum stem also contains significant amounts of hydroxycinnamic acid, such as *p*-coumaric acid (*p*CA, up to 35 mg/g cell walls), which is far higher than other agricultural waste biomass such as rice straw (6.8 mg/g cell walls) [21, 22]. *p*CA is widely used in the health and pharmaceutical industries because of a variety of physiological functions such as anti-inflammatory, antineoplastic, and antimicrobial activities [23]. Its phenolic acid structure can eliminate free radicals in the body and has a preventive effect on diseases closely related to oxidative stress damage [24]. *p*CA is also a precursor for the production of value-added aromatic chemical products [25]. In lignocellulose, *p*CA is attached to lignin via an ester linkage [26]. The ester bond can be broken under mild alkaline conditions, so mild alkali treatment was often used for extraction of *p*CA and more than 90% of *p*CA can be released from agricultural residues [8, 22].

Previous studies have reported the recalcitrance to enzymatic digestion in different parts of internodes from sugar cane due to cell type and chemical composition [27]. The sugar cane pith region contained parenchyma cells that were not extensively lignified. In contrast, the rind region contained highly lignified vessels and fibers and was very recalcitrant to enzymatic hydrolysis [27]. Similar trend was observed in sorghum stem. The sorghum stem parts can be divided into inner region and rind part, which have different lignin content, hydroxycinnamic acid content and carbohydrate composition [21]. Although it was frequently reported that organic solvent-assisted alkaline pretreatment can reduce over-degradation of cellulose and hemicelluloses [16, 28], the significance of organic solvent addition in NaOH pretreatment of different stem parts of sorghum is still unclear. In this study, NaOH–ethanol pretreatment was described to facilitate the release of *p*CA in different parts of sorghum stem, but minimize the degradation of sugar polymers (cellulose and hemicellulose) as much as possible, and therefore enhance the total reducing sugar recovery. The processing conditions (NaOH loading, ethanol content, processing time and temperature) were optimized using response surface methodology. Structural characterizations of raw and pretreated sorghum sample were investigated by Fourier transform infrared (FTIR) spectroscopy, scanning electron microscopy (SEM) and X-ray diffraction (XRD).

## Results and discussion

### Composition of raw sorghum samples

The composition of raw pith, rind and whole stem on a dry matter basis is shown in Table 1. The content of esterified *p*CA in pith was 2.21%, which was slightly higher than that in rind (2.08%). The content of cellulose (40.64%) and lignin (24.04%) in rind was slightly higher than that in pith (37.20% and 21.48%, respectively), while the xylan content of the rind (17.33%) was slightly lower than that of the pith (18.51%). The total carbohydrates including glucan, xylan, and arabinan in rind and pith reached 60.29% and 58.25%, respectively. It was worth noting that other components such as ash, extractives and lower content of sugars also exist in sorghum stems according to previous report [29], despite these constituents were not analyzed in this study. Variations in sorghum stem composition from previous researches were observed in this study, which may be due to the differences in the geographical location, fertilization, heterogeneity of feedstock of the samples and several other environmental factors [8, 29].

**Table 1:** Composition of raw sorghum pith, rind and the whole stem

	Glucan (%)	Xylan (%)	Arabinan (%)	Lignin (%)	Ester-linked *p*CA (%)	Ester-linked ferulic acid (%)
Pith	37.20 ± 0.19	18.51 ± 0.10	2.54 ± 0.06	21.48 ± 0.23	2.21 ± 0.14	0.38 ± 0.07
Rind	40.64 ± 0.08	17.33 ± 0.13	2.32 ± 0.11	24.04 ± 0.27	2.08 ± 0.22	0.43 ± 0.04
Whole stem	38.22 ± 0.21	17.70 ± 0.07	2.47 ± 0.09	23.35 ± 0.31	2.13 ± 0.17	0.41 ± 0.09

### Comparison of NaOH–ethanol pretreatment of different sorghum stem parts

Box–Behnken models were designed to optimize various process parameters (NaOH loading A, ethanol content B, temperature C and time D) in NaOH–ethanol pretreatment of different sorghum stem parts. The following equations were derived for the analysis of the release of *p*CA and the recovery of xylan in residues.

#### Pith

$$ \begin{aligned} p{\text{CA release yield }}\left( \% \right) & = + \; 7 9. 9 9+ 2 7. 4 6 {\text{A}} + 7.0 1 {\text{B}} + 4. 7 3 {\text{C}} + 5. 3 2 {\text{D}} + 1. 6 3 {\text{AB }} \\ & \quad - \; 4. 2 7 {\text{AC}} + 2. 7 1 {\text{AD}} + 1. 70{\text{BC}} - 7. 8 2 {\text{BD}} - 1. 1 3 {\text{CD}} - 1 5. 6 9 {\text{A}}^{ 2} - 2. 9 8 {\text{B}}^{ 2} - 3.00{\text{C}}^{ 2} - 0. 9 9 {\text{D}}^{ 2} \\ \end{aligned} $$

$$ \begin{aligned} {\text{Xylan recovery yield }}\left( \% \right) & = + \; 80. 3 9- 9. 8 8 {\text{A}} + 3. 2 6 {\text{B}} - 0. 3 5 {\text{C}} - 0. 4 3 9 2 {\text{D}} - 0. 5 4 {\text{AB }} \\ & \quad + \; 1. 1 1 {\text{AC}} - 0. 9 5 {\text{AD}} - 2. 8 3 {\text{BC}} + 4. 8 3 {\text{BD}} + 2. 4 8 {\text{CD}} - 3. 1 3 {\text{A}}^{ 2} - 1. 2 3 {\text{B}}^{ 2} - 2. 5 1 {\text{C}}^{ 2} - 2. 40{\text{D}}^{ 2} \\ \end{aligned} $$

#### Rind

$$ \begin{aligned} p{\text{CA release yield }}\left( \% \right) & = + \; 8 5. 5 2+ 3 3. 6 2 {\text{A}} + 6. 5 4 {\text{B}} + 3. 5 7 {\text{C}} + 7. 3 8 {\text{D}} + 5.0 3 {\text{AB }} \\ & \quad - \;0. 5 7 {\text{AC}} - 1. 2 7 {\text{AD}} - 0. 5 9 {\text{BC}} - 7. 9 2 {\text{BD}} - 1. 8 3 {\text{CD}} - 2 2. 4 8 {\text{A}}^{ 2} - 5. 1 4 {\text{B}}^{ 2} - 4. 2 2 {\text{C}}^{ 2} - 2. 50{\text{D}}^{ 2} \\ \end{aligned} $$

$$ \begin{aligned} {\text{Xylan recovery yield }}\left( \% \right) & = + \; 8 1. 2 4- 3. 5 1 {\text{A}} + 4. 3 3 {\text{B}} - 0. 5 4 4 2 {\text{C}} - 1. 5 6 {\text{D}} + 3.00{\text{AB }} \\ & \quad - \;0. 9 2 2 5 {\text{AC}} - 0.0 7 {\text{AD}} + 0. 9 4 5 {\text{BC}} - 1. 9 3 {\text{BD}} - 0. 4 1 {\text{CD}} \\ \end{aligned} $$

#### Whole stem

$$ \begin{aligned} p{\text{CA release yield }}\left( \% \right) & = + \;{ 82}.0 6+ 2 8. 9 2 {\text{A}} + 6. 6 7 {\text{B}} + 4. 7 9 {\text{C}} + 5. 4 4 {\text{D}} + 2. 9 3 {\text{AB }} \\ & \quad {-}\; 4. 3 4 {\text{AC}} + 1. 1 2 {\text{AD}} + 0. 4 8 7 5 {\text{BC}} - 7. 4 5 {\text{BD}} + 0. 9 3 {\text{CD}} - 1 6. 8 8 {\text{A}}^{ 2} - 3. 4 6 {\text{B}}^{ 2} - 2. 4 5 {\text{C}}^{ 2} - 1. 2 4 {\text{D}}^{ 2} \\ \end{aligned} $$

$$ \begin{aligned} {\text{Xylan recovery yield }}\left( \% \right) & = + \; 80. 3 9- 7. 2 3 {\text{A}} + 4. 2 7 {\text{B}} - 0. 1 70 8 {\text{C}} - 0. 5 6 {\text{D}} - 1. 9 6 {\text{AB }} \\ & \quad + \; 1. 6 5 {\text{AC}} - 0. 3 2 {\text{AD}} + 2. 1 4 {\text{BC}} - 1. 6 8 {\text{BD}} - 1. 6 9 {\text{CD}} - 5.0 8 {\text{A}}^{ 2} - 1. 20{\text{B}}^{ 2} + 0. 3 3 8 {\text{C}}^{ 2} - 0. 5 1 4 3 {\text{D}}^{ 2} \\ \end{aligned} $$

The effect of different variables on the release of *p*CA and the recovery of xylan in residues from different sorghum parts were investigated. Overall, NaOH concentration and ethanol content were the first and second most influential factors on the *p*CA release yield and xylan recovery yield, respectively. As shown in Table 2, the released contents of *p*CA from the sorghum stem were strongly depended on the NaOH concentration; however, some variation existed among different stem parts. At a low NaOH concentration (i.e., 0.5%), the release of *p*CA was very low, but significantly higher *p*CA was released from the pith than from the rind under the same conditions. As the NaOH concentration increased and the ethanol content exceeded 40% (v/v), the release of *p*CA from the rind slightly exceeded over that from the pith under the same conditions. NaOH concentration is also the key factor affecting the xylan dissolution, and more xylan was dissolved as NaOH concentration increased. In general, xylan was more resistant to be dissolved from the rind than from the pith in the same NaOH–ethanol conditions. This may be related to the difference in cell types and chemical composition in different sorghum stem parts. The pith is rich in parenchyma cells and the rind contains more vascular bundles. Parenchyma cells in pith have bigger lumens and thinner cell walls, and vascular bundles in rind are composed of tightly packed vessel elements [30]. On other hand, the easier dissolution of xylan from pith than those from rind under the same conditions may be partly attributed to the lower lignin content in the sorghum pith than in the rind [27]. Interestingly, there was a low linear correlation between the release of *p*CA and the recovery of xylan in residues for both pith and rind (Additional file 1: Fig. S1). Indeed, there is little chemically structural connection between *p*CA and xylan as most of *p*CA is attached to lignin with ester bond [31].

**Table 2:** Factors (NaOH loading, ethanol content, temperature, and time) and the responses (released *p*CA and recovered xylan in residues) of the Box–Behnken design used for response surface methodology

Run	Factor				Response					
	NaOH (%)	Ethanol (%)	Temperature (°C)	Time (h)	Released *p*CA (%)			Recovered xylan in residues (%)		
					Pith	Rind	Whole	Pith	Rind	Whole
1	1.25	10	70	2.5	66.23	67.64	67.57	79.78	74.35	75.21
2	1.25	70	65	1	83.99	83.74	83.82	76.06	86.71	84.69
3	1.25	40	65	2.5	79.66	85.56	81.68	80.42	82.21	80.28
4	2	10	65	2.5	85.05	88.45	88.29	63.95	72.78	62.18
5	1.25	40	60	4	79.22	86.40	79.70	73.22	81.10	82.49
6	1.25	10	65	4	84.90	87.36	87.19	65.39	77.34	77.80
7	1.25	70	60	2.5	75.00	82.14	78.72	81.84	84.69	79.63
8	2	40	65	1	85.44	88.31	87.60	65.11	80.53	70.17
9	0.5	40	70	2.5	45.47	25.97	45.02	79.98	82.44	81.01
10	0.5	40	65	4	32.36	31.59	33.02	89.17	82.95	80.13
11	1.25	40	65	2.5	80.27	84.54	82.89	81.03	82.07	81.02
12	0.5	70	65	2.5	36.49	21.49	32.92	88.92	85.44	88.17
13	2	40	60	2.5	86.89	92.30	90.52	64.88	79.12	68.76
14	1.25	70	65	4	84.15	86.52	85.85	83.71	82.64	81.43
15	0.5	10	65	2.5	33.08	32.00	34.48	82.73	85.31	73.70
16	1.25	70	70	2.5	90.14	91.14	90.73	79.94	86.96	87.58
17	1.25	40	65	2.5	78.96	84.25	82.47	80.76	83.11	79.87
18	0.5	40	65	1	31.53	19.42	29.79	85.12	89.10	83.22
19	2	40	70	2.5	87.14	93.68	90.17	64.70	74.80	70.11
20	2	70	65	2.5	94.97	98.07	98.44	67.98	84.89	68.77
21	1.25	40	65	2.5	79.28	87.44	80.13	79.82	82.89	80.05
22	1.25	10	60	2.5	57.88	56.28	57.51	70.35	75.86	75.80
23	1.25	40	60	1	67.08	66.70	71.29	80.95	83.15	78.85
24	0.5	40	60	2.5	28.14	22.31	28.02	84.60	83.07	86.24
25	1.25	10	65	1	53.48	52.89	55.36	77.08	73.69	74.36
26	1.25	40	65	2.5	81.77	85.79	83.11	79.91	82.58	80.74
27	1.25	40	70	4	84.80	91.44	90.94	74.72	79.11	76.34
28	1.25	40	70	1	77.19	79.06	78.81	72.52	82.80	79.47
29	2	40	65	4	97.12	95.41	95.30	65.36	74.10	65.80

The regression coefficients of the model were determined by analysis of variance (ANOVA) in Additional file 1: Table S1. All models were significant at the *P* < 0.0001 level, which shows that all models were valid and do not lack fit, so this indicates that the model can be used to predict response. Several optimized solutions for model prediction were selected based upon the constraints: maximum xylan recovery in residues from different sorghum stem parts with the prerequisite of approximately 95% *p*CA release yield (not maximized). As shown in Table 3, the actual value was close to predicted value, which verified the reliability of the model. Compared with pith, the NaOH concentration in rind was higher, and the treatment time was shorter. The possible reason is that the structure of the rind is denser, so higher NaOH concentration was needed to release *p*CA, and in turn shortening the processing time.

**Table 3:** Confirmation of the predicted optimum condition with the experimental results

NaOH (%)	Ethanol (%)	Temperature (°C)	Time (h)	Released *p*CA (%)		Recovered xylan in residues (%)	
				Predicted values	Actual values	Predicted values	Actual values
Pith							
1.63	70	66.0	3.18	94.59	94.07	77.28	76.85
1.72	70	63.4	2.29	94.00	94.22	76.20	76.01
1.51	70	65.2	3.30	92.63	91.76	77.69	76.32
Rind							
1.90	70	69.8	1.00	98.99	97.24	88.73	88.46
1.80	70	62.1	1.50	97.28	97.05	87.56	86.47
1.55	70	68.8	1.95	96.00	94.74	86.79	84.99
Whole stem							
1.46	70	70.0	2.19	95.13	95.05	85.35	85.04
1.52	70	70.0	2.00	95.90	94.77	83.68	82.96
1.39	70	70.0	3.00	92.07	92.31	82.85	82.12

**Fig. 1:**
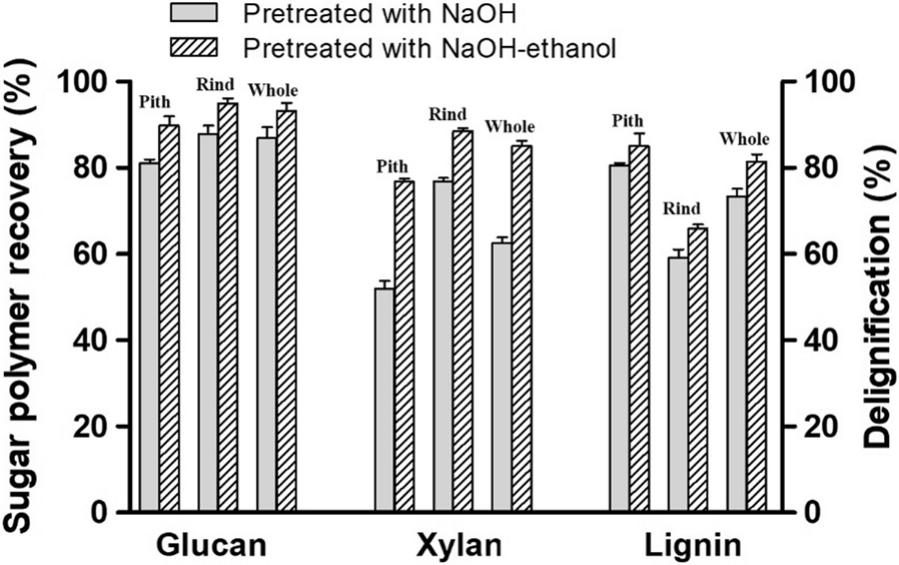
Sugar recovery (glucan and xylan) and lignin removal after pretreatment. NaOH–ethanol pretreatment was performed under the following conditions: 1.63% NaOH, 70% ethanol, 66 °C, 3.18 h; 1.90% NaOH, 70% ethanol, 69.8 °C, 1.00 h; and 1.46% NaOH, 70% ethanol, 70 °C, 2.19 h for pith, rind and whole stem, respectively. NaOH pretreatment was performed under the same conditions, except that 70% ethanol was not included

According to the above optimization using response surface methodology, the following conditions were selected for NaOH–ethanol pretreatment to obtain the maximum release of *p*CA and the maximum recovery of xylan in residues for the pith, rind and whole stem in further experiments: 1.63% NaOH, 70% ethanol, 66 °C, 3.18 h; 1.90% NaOH, 70% ethanol, 69.8 °C, 1.00 h and 1.46% NaOH, 70% ethanol, 70 °C, 2.19 h, respectively. NaOH pretreatment was also carried out in same conditions (except ethanol) to further understand the significance of ethanol in NaOH–ethanol pretreatment. The solid recovery, *p*CA release and content of each component in solid residues are shown in Table 4. In general, the solid recovery, *p*CA release and the glucan and xylan content of NaOH–ethanol-pretreated residues were relatively higher than the corresponding NaOH-pretreated residues, but the lignin content was lower than the NaOH-pretreated residues. These results suggested that ethanol addition promoted the release of *p*CA and delignification to a certain extent, but obviously prevented the deconstruction of xylan and cellulose. Compared to NaOH pretreatment, the release yields of *p*CA in pith, rind and whole stem by NaOH–ethanol pretreatment were increased by 8.16%, 8.38% and 8.39%, respectively (Table 4). Meanwhile, the delignification rates were increased by 5.57%, 11.47% and 11.04%, respectively (Fig. 1). The glucan recoveries of pith, rind and whole stem after NaOH–ethanol pretreatment reached 89.78%, 94.85% and 93.19%, which increased by 10.76%, 7.97% and 7.29% compared to NaOH pretreatment. The xylan recoveries of pith, rind and whole stem after NaOH–ethanol pretreatment reached 76.80%, 88.46% and 85.01%, which increased by 47.75%, 15.11% and 35.97% compared to NaOH pretreatment (Fig. 1). Despite more delignification by NaOH–ethanol pretreatment, the solid recovery of the NaOH–ethanol-pretreated pith, rind and whole stem were 6.71%, 3.31% and 6.50% higher than those of NaOH pretreatment, respectively, which was mainly due to the improved recovery of glucan and xylan (Table 4). Particularly, the dissolution of xylan in pith was more serious in NaOH pretreatment condition compared to NaOH–ethanol pretreatment, indicating that addition of ethanol in NaOH solution could effectively minimize the deconstruction of xylan in pith (Fig. 1). Overall, the addition of ethanol to the NaOH pretreatment released almost all of esterified *p*CA while retaining most of cellulose and hemicellulose in the solid residue.

**Table 4:** The release of *p*CA during pretreatment, the recovery of the solid residue and the content of each component in solid residues

Materials	Solid recovery (%)	Released *p*CA (%)	Composition			
			Glucan (%)	Xylan (%)	Arabinan (%)	Lignin (%)
NaOH–ethanol						
Pith	59.48 ± 0.54	94.07 ± 1.34	56.15 ± 0.72	23.90 ± 0.27	2.95 ± 0.14	5.40 ± 0.16
Rind	75.85 ± 0.71	97.24 ± 2.11	50.82 ± 0.17	20.21 ± 0.93	2.45 ± 0.07	10.81 ± 0.09
Whole stem	68.52 ± 0.59	95.05 ± 1.57	51.98 ± 1.04	21.96 ± 0.12	2.73 ± 0.20	6.32 ± 0.21
NaOH						
Pith	55.74 ± 1.16	86.97 ± 0.79	54.10 ± 0.35	17.26 ± 1.12	2.64 ± 0.23	7.49 ± 1.06
Rind	73.42 ± 0.78	89.72 ± 1.96	48.63 ± 1.86	18.14 ± 0.89	2.12 ± 0.11	13.39 ± 0.95
Whole stem	64.34 ± 0.94	87.69 ± 1.63	51.60 ± 1.03	17.21 ± 0.67	2.34 ± 0.18	9.67 ± 0.64

NaOH–ethanol pretreatment was performed under the following conditions: 1.63% NaOH, 70% ethanol, 66 °C, 3.18 h; 1.90% NaOH, 70% ethanol, 69.8 °C, 1.00 h and 1.46% NaOH, 70% ethanol, 70 °C, 2.19 h for the pith, rind and whole stem, respectively. NaOH pretreatment was performed under the same conditions, except that 70% ethanol was not included

The inhibitors including acetic acid, levulinic acid, furan (furfural and 5-hydroxymethyl furfural (HMF)) and ferulic acid in pretreated liquid fractions were analyzed. The data showed that acetic acid and ferulic acid were the main inhibitors produced during pretreatments (Table 5). In comparison, the ferulic acid released in the NaOH–ethanol-pretreated liquid fraction was slightly more than that in the NaOH-pretreated liquid fraction, but the content of acetic acid was less than that in the NaOH-pretreated liquid fraction. No levulinic acid, furfural and HMF were detected in both pretreated liquid fractions.

**Table 5:** Inhibitors produced in liquid fractions during the pretreatment of sorghum pith, rind and whole stem

Materials	Ferulic acid (g/L)	Acetic acid (g/L)	Levulinic acid (g/L)	Furfural (g/L)	HMF (g/L)
NaOH–ethanol					
Pith	0.184	1.428	n.d.	n.d.	n.d.
Rind	0.210	1.448			
Whole stem	0.204	1.490			
NaOH					
Pith	0.183	1.462	n.d.	n.d.	n.d.
Rind	0.194	1.487			
Whole stem	0.186	1.521			

NaOH–ethanol pretreatment was performed under the following conditions: 1.63% NaOH, 70% ethanol, 66 °C, 3.18 h; 1.90% NaOH, 70% ethanol, 69.8 °C, 1.00 h and 1.46% NaOH, 70% ethanol, 70 °C, 2.19 h for the pith, rind and whole stem, respectively. NaOH pretreatment was performed under the same conditions, except that 70% ethanol was not included

*n.d.* not detected

### Enzymatic hydrolysis of solid residues by Cellic^®^ CTec2

**Fig. 2:**
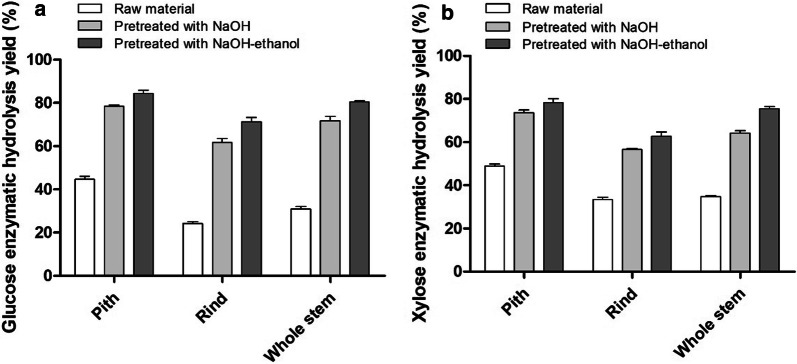
Glucose (**a**) and xylose (**b**) enzymatic hydrolysis yields of raw and pretreated materials. The sample (2%, w/v) was hydrolyzed at 50 °C for 72 h using Cellic^®^ CTec2 (15 FPU/g substrate) and β-glucosidase (30 CBU/g substrate)

The enzymatic hydrolysis yields of raw and pretreated materials with Cellulase Cellic^®^ CTec2 and β-glucosidase are shown in Fig. 2. The glucose enzymatic hydrolysis yields of NaOH–ethanol-pretreated pith, rind and whole stem were 84.29%, 71.22% and 80.48%, respectively, which were 88.95%, 194.54% and 160.20% higher than that of untreated pith, rind and whole stem, respectively (Fig. 2a). Similarly, the xylose enzymatic hydrolysis yields of NaOH–ethanol-pretreated pith, rind and whole stem were 78.33%, 62.70% and 75.53%, respectively, which were 59.89%, 87.78% and 117.23% higher than untreated pith, rind and whole stem, respectively (Fig. 2b). The enzymatic hydrolysis efficiency of the NaOH–ethanol-pretreated residues was also obviously higher than that of NaOH-pretreated residues. The glucose enzymatic hydrolysis yields of NaOH–ethanol-pretreated pith, rind and whole stem were 7.39%, 15.45% and 12.18% higher than that of the NaOH-pretreated pith, rind and whole stem, respectively (Fig. 2a). The xylose enzymatic hydrolysis yields of NaOH–ethanol-pretreated pith, rind and whole stem were 6.43%, 10.86% and 17.61% higher than NaOH-pretreated pith, rind and whole stem, respectively (Fig. 2b). This may be attributed to more effective in delignification by NaOH–ethanol pretreatment compared to that of NaOH pretreatment, because lignin is one of the major factors inhibiting enzymatic saccharification [32]. This result was consistent with the previous finding reported by Huang et al. [28]. They described that the introduction of ethanol into alkaline peroxide pretreatment enhanced the delignification of bamboo and thus improved its enzymatic hydrolysis efficiency. In addition, the improvement of the enzymatic saccharification efficiency of the NaOH–ethanol-pretreated residues might be also partly brought by its higher release of *p*CA compared to NaOH pretreatment, since it has been reported that the presence of phenolic acids in residues has a negative effect on the enzymatic hydrolysis of lignocellulose [33]. In summary, alkali organosolv pretreatment not only reduces the over-degradation of cellulose and hemicellulose, but also enhances the delignification of biomass, resulting in enhanced enzymatic digestion of biomass [34, 35].

### Effect of xylanase on enzymatic hydrolysis of solid residues by Cellic^®^ CTec2

**Fig. 3:**
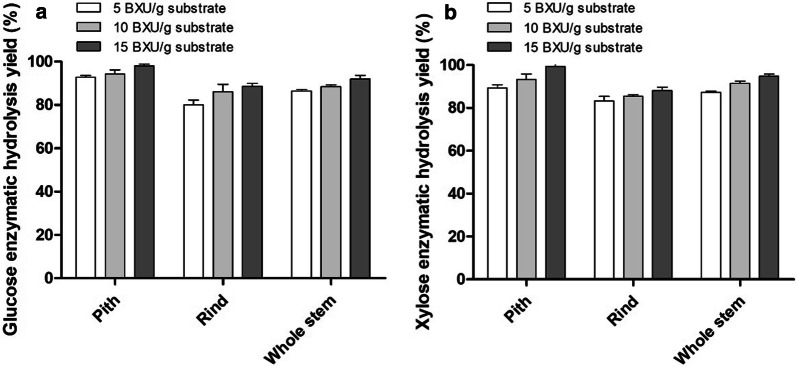
Effect of different dosages of adding xylanase on enzymatic hydrolysis of NaOH–ethanol-pretreated materials. The sample (2%, w/v) was hydrolyzed at 50 °C for 72 h using cellulose cocktail (15 FPU/g substrate Cellic^®^ CTec2, 30 CBU/g substrate β-glucosidase and 5-15 BXU/g substrate xylanase)

**Fig. 4:**
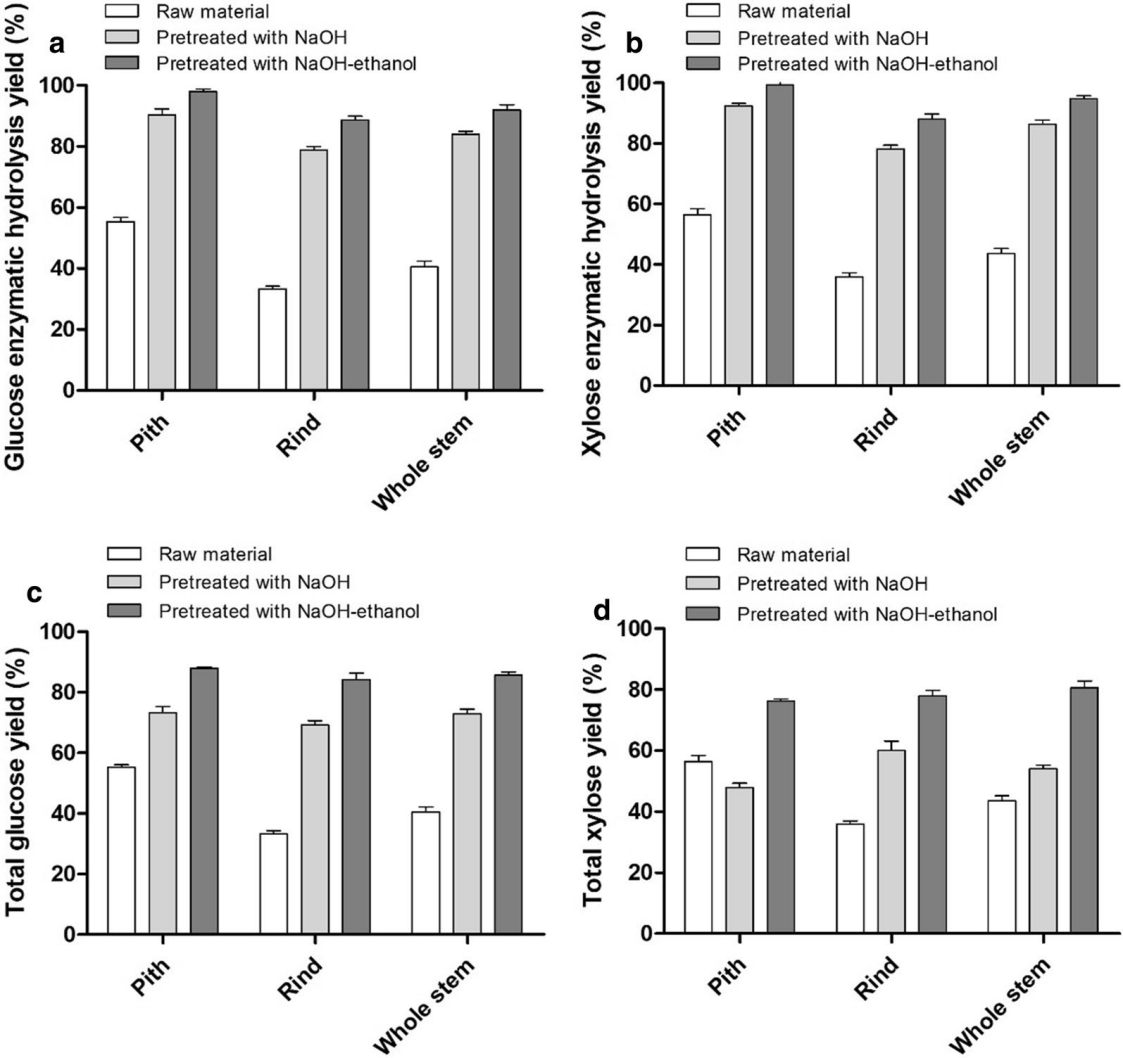
Comparison of enzymatic hydrolysis yields [glucose (**a**) and xylose (**b**)] and total yields of sugar [glucose (**c**) and xylose (**d**)] of raw, NaOH-pretreated and NaOH–ethanol-pretreated materials with adding xylanase. The sample (2%, w/v) was hydrolyzed at 50 °C for 72 h using cellulose cocktail (15 FPU/g substrate Cellic^®^ CTec2, 30 CBU/g substrate β-glucosidase and 15 BXU/g substrate xylanase)

Since relatively high xylan was retained in NaOH–ethanol-pretreated residues, the saccharification of cellulose may be still impeded by the highly reserved xylan in the lignocellulosic matrix [36]. Therefore, the effect of xylanase (Sigma X2629-100g) on enzymatic hydrolysis of solid residues was studied by adding xylanase to enzymatic hydrolysis system. Three different dosages of xylanase (5 BXU, 10 BXU and 15 BXU/g substrate) were selected for the enzymatic hydrolysis of NaOH–ethanol-pretreated solid residues, and the results are shown in Fig. 3. After the addition of xylanase, the enzymatic hydrolysis yields of glucose and xylose were gradually improved with the increase of xylanase dosage (Fig. 3). This was attributable to the increased accessibility of cellulose to cellulase when xylan was degraded by xylanase. As shown in Fig. 4, when the dosage of xylanase was 15 BXU/g substrate, the glucose enzymatic hydrolysis yields of NaOH–ethanol-pretreated pith, rind and whole stem reached 97.93%, 88.71% and 91.98%, respectively, which increased by 16.18%, 24.56% and 14.29% compared to those without xylanase (Fig. 4a), and the xylose enzymatic hydrolysis yields also reached 99.34%, 88.11% and 94.79% (Fig. 4b), which increased by 26.82%, 40.53% and 25.50% compared to those without xylanase. Similarly, the glucose enzymatic hydrolysis and xylose enzymatic hydrolysis yields were also increased when xylanase was added during the enzymatic hydrolysis of NaOH-pretreated sample. The glucose enzymatic hydrolysis yields of the NaOH-pretreated pith, rind and whole stem reached 90.36%, 78.86% and 84.00%, respectively, which increased by 15.12%, 27.83% and 17.09% compared to those without xylanase, and the xylose enzymatic hydrolysis yields were enhanced to 92.43%, 78.22% and 86.41%, respectively, which increased by 25.58%, 38.30% and 34.55% compared to those without xylanase (Fig. 4a, b). These results confirmed the promoting effect of xylanase on enzymatic hydrolysis of solid residues, which was consistent with the previous report of negative effects of retaining xylan on enzymatic hydrolysis of cellulose [37]. In comparison, the percentage increases in enzymatic saccharification by adding xylanase are more significant in rind than pith, probably due to higher recovered xylan in rind.

The total_Glu_. yields and total_Xyl_. yields of various pretreated materials after adding xylanase (15 BXU/g substrate) were calculated. In above condition, the total_Glu_. yields of NaOH–ethanol-pretreated pith, rind and whole stem were 87.92%, 84.14% and 85.72%, respectively (Fig. 4c), which were 20.03%, 21.45% and 17.49%, respectively, higher than those of NaOH-pretreated samples. The total_Xyl_. yields were 76.29%, 77.94% and 80.58% (Fig. 4d), which were 58.77%, 29.66% and 49.17%, respectively, higher than those of NaOH-pretreated samples. Taken together, the total reducing sugar yields (glucose and xylose) after enzymatic hydrolysis reached 84.06%, 82.29% and 84.09% for NaOH–ethanol-pretreated pith, rind and whole stem, respectively, which were 29.56%, 23.67% and 25.56% higher than the corresponding NaOH-pretreated samples. The substantial increase of total reducing sugar yields was attributed to the improvement in both solid recovery and enzymatic hydrolysis yields when using NaOH–ethanol pretreatment. It was worth noting that the total_Xyl_. yield of the NaOH-pretreated pith was lower than that of the untreated pith, because nearly half of the xylan was degraded during the NaOH pretreatment.

### Structural characterization of untreated and pretreated sorghum sample

#### FTIR spectrum analysis

The aromatic structure strength and functional group identification of the untreated and treated samples were investigated using FTIR techniques. FTIR spectra of the untreated and treated samples are shown in Additional file 1: Fig. S2. The wavelength assignments of the lignin-, cellulose- and hemicellulose-related bands are summarized in Additional file 1: Table S2. Absorption spectra at 1732/cm are due to the ester-linked acetyl, feruloyl and *p*-coumaroyl groups on hemicellulose and/or lignin [38]. The band of the pretreated material at 1732/cm almost completely disappeared, indicating that almost all of the ester-linked phenolic acid were released into the liquid. The bands at 1106/cm, 1254/cm and 1513/cm are characteristic bands of lignin [39]. These lignin-related bands were significantly reduced after treatment, indicating that most of the lignin was removed during pretreatment. After pretreatment, the characteristic band of the β-anomer or the β-linked glucose polymer increased significantly at 899/cm, indicating a significant increase in the cellulose content in the treated residue [40]. In summary, FTIR spectroscopy results show that the pretreatment can break the ester bond of *p*CA to lignin and remove most of the lignin.

#### SEM analysis

To observe the changes in the substrate surface, SEM was applied to investigate the morphological features and surface characteristics of the raw and the pretreated pith, rind and whole stem. As can be seen from Additional file 1: Fig. S3, the surface of the raw material was relatively smooth. The surfaces of the pretreated residue were altered with evident coarse surface and porous areas. Available surface area of the cellulose fiber structure is essential for enzymatic hydrolysis of lignocellulosic materials [41]. Both NaOH–ethanol and NaOH pretreatment destroyed the recalcitrant structure of the lignocelluloses, increased the surface area and porosity of biomass, which accelerated the saccharification process.

#### XRD analysis

The crystallinity index (CrI) was calculated according to X-ray diffractograms. XRD analysis of untreated and pretreated sorghum samples is shown in Additional file 1: Fig. S4. In raw materials, the CrI of the pith, rind and whole stem were 30.29%, 34.61% and 30.84%, respectively. After NaOH–ethanol pretreatment, the CrI of pith, rind and whole stem were increased to 63.01%, 43.27% and 54.41%, respectively. Similarly, the CrI of NaOH-pretreated pith, rind and whole stem were also increased to 58.14%, 42.17% and 45.86%, respectively. Lignocellulosic crystallinity was considered as an important characteristic for enzymatic digestibility [42]. According to previous reports, the CrI of lignocelluloses was inversely related to the amorphous substances in cell wall where degradation of hemicelluloses and disordered fractions of cellulose, and delignification can all make the CrI increase [36]. Therefore, higher CrI of NaOH–ethanol-pretreated residue may be due to the higher level of lignin removal and the recovery of cellulose compared to NaOH-pretreated residue.

### Overall mass balance

**Fig. 5:**
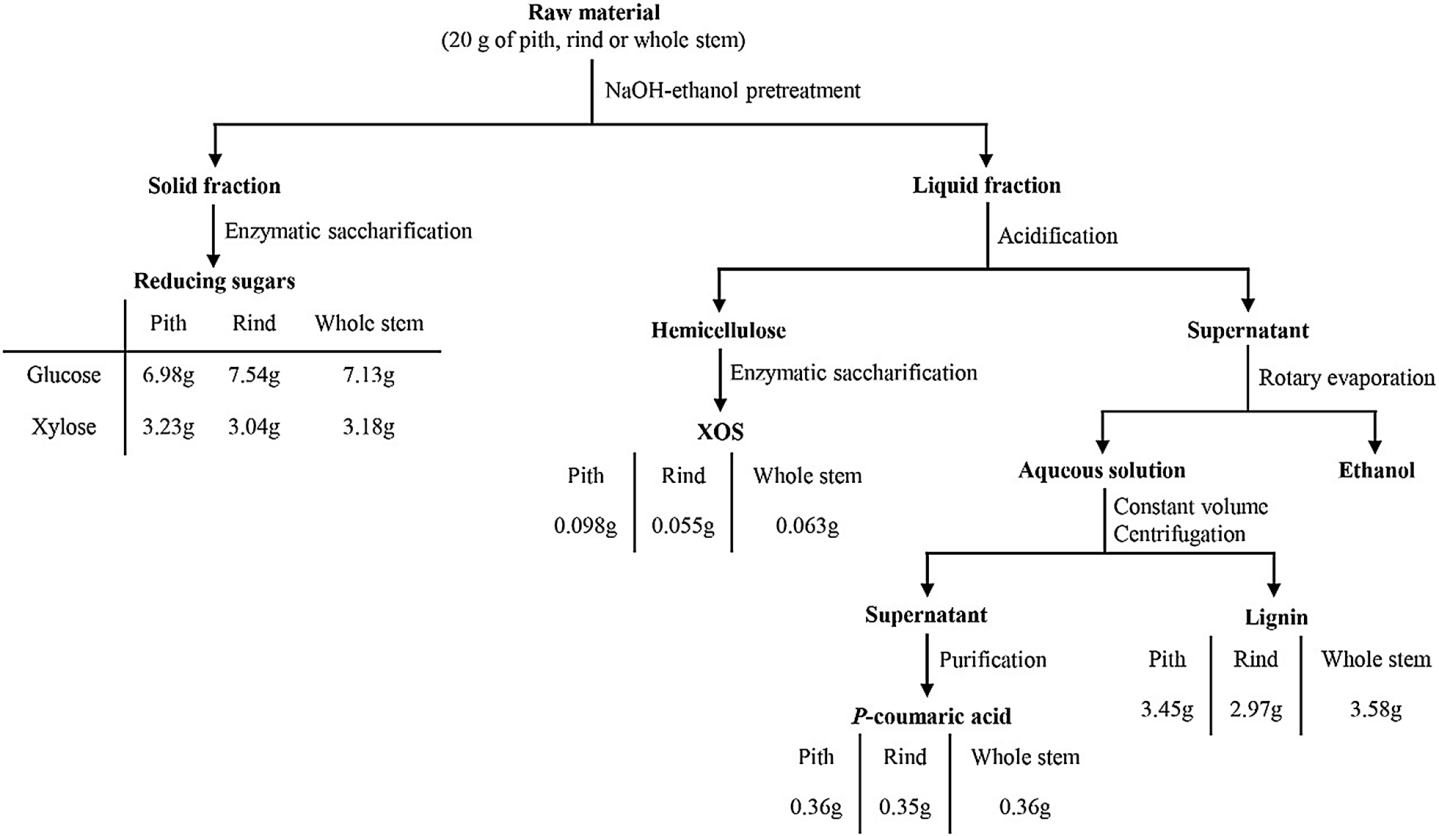
Overall mass balance for the process of NaOH–ethanol pretreatment and enzymatic saccharification. The overall mass balance was investigated with 20 g of the each raw material (pith, rind or whole stem). The conditions of pretreatment were as follows: 1.63% NaOH, 70% ethanol, 66 °C, 3.18 h; 1.90% NaOH, 70% ethanol, 69.8 °C, 1.00 h, and 1.46% NaOH, 70% ethanol, 70 °C, 2.19 h for pith, rind and whole stem, respectively. The solid residues were enzymatically hydrolyzed with cellulose cocktail (15 FPU/g substrate Cellic^®^ CTec2, 30 CBU/g substrate β-glucosidase and 15 BXU/g substrate xylanase)

The process of pretreatment and enzymatic saccharification of sorghum samples (20 g) is shown in Fig. 5. NaOH–ethanol pretreatment of the pith, rind and whole stem was performed under the respective optimal conditions. Enzymatic saccharification of pretreated pith, rind and whole stem yielded 6.98 g, 7.54 g and 7.13 g of glucose, respectively, which accounted for 84.37%, 83.46% and 84.02% of the glucose in sorghum pith, rind and whole stem, respectively. Meanwhile, enzymatic saccharification produced 3.23 g, 3.04 g and 3.18 g of xylose, respectively, which accounted for 76.72%, 77.21% and 79.16% of the xylose in sorghum pith, rind and whole stem, respectively. After acidification, the NaOH–ethanol-pretreated liquid fractions of pith, rind and whole stem contained 0.41 g, 0.40 g and 0.40 g of *p*CA, respectively. Macroporous adsorption resin D101 was used to recover *p*CA, and the adsorption rate and desorption rate reached 96% and 91%, respectively. After desorption from resin column, the recovered *p*CA were 0.36 g, 0.35 g and 0.36 g, which accounted for 81.45%, 84.13% and 84.51% of the esterified *p*CA in sorghum pith, rind and whole stem, respectively. Since the macroporous adsorption resin also has an adsorption capacity on other hydroxycinnamic acid derivatives such as ferulic acid in liquid fraction, further purification procedure will be needed to obtain high purity of *p*CA. In this study, optimization using the response surface methodology revealed that the pith, rind and whole stem require different NaOH–ethanol pretreatment conditions for maximal *p*CA release and xylan preservation due to the difference in cell type and chemical composition of the pith and rind. From the perspective of *p*CA release yield and total sugar yield, however, there were no huge differences between the pith and rind if they were pretreated by respective optimal NaOH–ethanol conditions. Considering the separation cost of the different stem parts, whole sorghum stem can be directly used as feedstock for biorefinery.

Moreover, the hemicellulose obtained from the liquid fractions of the pretreated pith, rind, and whole stem was 0.74 g, 0.38 g and 0.51 g, respectively, which contained 42.64%, 44.29% and 40.97% of xylan, respectively. The hemicellulose was collected and subjected to enzymatic hydrolysis for production of xylooligosaccharides (XOS). EpXYN1 displayed the best enzymatic hydrolysis efficiency, and the total XOS (xylobiose to xylohexaose) yields based on xylan from the pretreated pith, rind and whole stem were 31.19%, 32.71% and 30.28%, respectively (Additional file 1: Table S3). The results suggested that the dissolved xylan during the NaOH–ethanol pretreatment process can also be utilized for XOS production.

## Conclusions

In this study, NaOH–ethanol pretreatment was used to treat different parts of the sorghum (pith and rind) to enhance the release of *p*CA and the recovery of total reducing sugars. The pith, rind and whole stem required different pretreatment conditions due to structure chemical composition differences. Under optimized conditions, the rind acquired higher *p*CA release and xylan recovery than the pith. There was no significant difference in the total sugar yield of different stem parts by respective optimal NaOH–ethanol pretreatment, so whole sorghum stem can be directly used as feedstock for biological refining in industrial production. Compared to NaOH pretreatment, higher release of *p*CA and recovery of hemicellulose were obtained in NaOH–ethanol pretreatment. The total reducing sugar yield was enhanced after NaOH–ethanol pretreatment and enzymatic hydrolysis because of the improvement of enzymatic hydrolysis and sugar polymers (glucan and xylan) recovery. All the results suggested that NaOH–ethanol pretreatment is a promise pretreatment method of sorghum biorefinery for product fermentable sugars and value-added products.

## Materials and methods

### Materials

Sorghum stem was obtained from Hengshui, Hebei Province, China. The pith and rind were separated by manual peeling with a sharp knife and washed three times with tap water to remove field dirt. After drying at 65 °C, sorghum samples were ground and passed through 40-mesh sieve. Cellulase Cellic^®^ CTec2 (89 FPU/g) and β-glucosidase (Novozyme 188, 300 CBU/mL) were purchased from Novozymes China (Shanghai, China). Commercial xylanase (X2629-100g, 470 BXU/g) was purchased from Sigma-Aldrich (Shanghai, China). The xylanase EpXYN1 (130 BXU/mL), EpXYN3 (722 BXU/mL) and XynII (546 BXU/mL) were prepared according to our previous researches [43, 44]. Other chemicals and reagents used in this study were of analytical grade.

### NaOH–ethanol pretreatment of different sorghum stem parts

The NaOH–ethanol pretreatment experiments were carried out with different temperature (60–70 °C) and time (1–4 h) in a plastic centrifuge tube. One gram of sorghum stem was suspended in 20 mL of mixed solution of NaOH (0.5–2%, w/v) and ethanol (10–70%, w/v). Addition of NaHSO_3_ (100 mg/L) prevents oxidation of the phenolic acid. The whole slurry was separated by vacuum filtration and washed by equal volume of water, then the liquids were combined. The treated solid was washed with water until the filtrate reached a neutral pH and then dried at 65 °C for the following experiments. The pH of NaOH–ethanol-extracted liquid fraction was adjusted to 1–2 with HCl, and then 1.5 times the volume of absolute ethanol was added to precipitate hemicellulose and allowed to stand at 4 °C for 24 h. The precipitate was collected as extracted xylan by centrifugation and dried at 65 °C for the following experiments. The liquid fraction was rotary evaporated to recover ethanol, and then the *p*CA was analyzed by high-performance liquid chromatography (HPLC, Agilent 1260, USA).

The solid recovery, *p*CA release, xylan recovery, glucan recovery, and delignification were calculated according to the following equations, respectively:

1 $$ {\text{Solid}}\;{\text{recovery}}\;{\text{yield }}\left( \% \right) = 100 \times {{\text{Regenerated residue}} \mathord{\left/ {\vphantom {{\text{Regenerated residue}} {\text{Raw material}}}} \right. \kern-0pt} {\text{Raw material}}} $$

2 $$ p{\text{CA}}\;{\text{release}}\;{\text{yield }}\left( \% \right) = 100 \times {{{\text{Amount}}\;{\text{of}}\;{\text{release}}\;p{\text{CA}}\;{\text{after}}\;{\text{pretreatment}}} \mathord{\left/ {\vphantom {{{\text{Amount}}\;{\text{of}}\;{\text{release}}\;p{\text{CA}}\;{\text{after}}\;{\text{pretreatment}}} {{\text{Amount}}\;{\text{of}}\;p{\text{CA}}\;{\text{in}}\;{\text{raw}}\;{\text{material}}}}} \right. \kern-0pt} {{\text{Amount}}\;{\text{of}}\;p{\text{CA}}\;{\text{in}}\;{\text{raw}}\;{\text{material}}}} $$

3 $$ {\text{Xylan}}\;{\text{recovery}}\;{\text{yield }}\left( \% \right) = 100 \times {{\left( {0. 8 8\times {\text{Amount}}\;{\text{of}}\;{\text{xylose}}\;{\text{in}}\;{\text{the}}\;{\text{residue}}} \right)} \mathord{\left/ {\vphantom {{\left( {0. 8 8\times {\text{Amount}}\;{\text{of}}\;{\text{xylose}}\;{\text{in}}\;{\text{the}}\;{\text{residue}}} \right)} {{\text{Amount}}\;{\text{of}}\;{\text{xylan}}\;{\text{in}}\;{\text{raw}}\;{\text{material}}}}} \right. \kern-0pt} {{\text{Amount}}\;{\text{of}}\;{\text{xylan}}\;{\text{in}}\;{\text{raw}}\;{\text{material}}}} $$

4 $$ {\text{Glucan}}\;{\text{recovery}}\;{\text{yield}}\;\left( \% \right){ = 1}00 \times {{\left( {0. 9\times {\text{Amount}}\;{\text{of}}\;{\text{glucose}}\;{\text{in}}\;{\text{residue}}} \right)} \mathord{\left/ {\vphantom {{\left( {0. 9\times {\text{Amount}}\;{\text{of}}\;{\text{glucose}}\;{\text{in}}\;{\text{residue}}} \right)} {{\text{Amount}}\;{\text{of}}\;{\text{glucan}}\;{\text{in}}\;{\text{raw}}\;{\text{material}}}}} \right. \kern-0pt} {{\text{Amount}}\;{\text{of}}\;{\text{glucan}}\;{\text{in}}\;{\text{raw}}\;{\text{material}}}} $$

5 $$ {\text{Delignification}}\;{\text{yield}}\;\left( \% \right) = 100 \times {{\left( {{\text{Amount}}\;{\text{of}}\;{\text{lignin}}\;{\text{in}}\;{\text{raw}}\;{\text{material}} - {\text{amount}}\;{\text{of}}\;{\text{lignin}}\;{\text{in}}\;{\text{residue}}} \right)} \mathord{\left/ {\vphantom {{\left( {{\text{Amount}}\;{\text{of}}\;{\text{lignin}}\;{\text{in}}\;{\text{raw}}\;{\text{material}} - {\text{amount}}\;{\text{of}}\;{\text{lignin}}\;{\text{in}}\;{\text{residue}}} \right)} {{\text{Amount}}\;{\text{of}}\;{\text{lignin}}\;{\text{in}}\;{\text{raw}}\;{\text{material}}}}} \right. \kern-0pt} {{\text{Amount}}\;{\text{of}}\;{\text{lignin}}\;{\text{in}}\;{\text{raw}}\;{\text{material}}}} $$

### Enzymatic hydrolysis

#### Enzymatic hydrolysis of solid residue by Cellic^®^ CTec2 with or without xylanase

The pretreated residue was enzymatically hydrolyzed in a conical flask with phosphate buffer (pH 4.8), and the substrate was added at 2% (w/v). Sodium azide (10 mM) was also added to the solution to prevent microbial contamination. The enzymatic hydrolysis was conducted at 50 °C for 72 h in the presence of Cellic^®^ CTec2 cellulase (15 FPU/g substrate), β-glucosidase (30 CBU/g substrate) with/without xylanase (Sigma X2629-100 g) in a shaking incubator at 150 rpm.

The glucose enzymatic hydrolysis yield, xylose enzymatic hydrolysis yield, total glucose yield, and total xylose yield were calculated according to the equations as follows:

6 $$ {\text{Glucose}}\;{\text{enzymatic}}\;{\text{hydrolysis}}\;{\text{yield}}\;\left( \% \right) = 100 \times {{\left( {0. 9\times {\text{Amount}}\;{\text{of}}\;{\text{released}}\;{\text{glucose}}\;{\text{after}}\;{\text{enzymatic}}\;{\text{hydrolysis}}} \right)} \mathord{\left/ {\vphantom {{\left( {0. 9\times {\text{Amount}}\;{\text{of}}\;{\text{released}}\;{\text{glucose}}\;{\text{after}}\;{\text{enzymatic}}\;{\text{hydrolysis}}} \right)} {{\text{Amount}}\;{\text{of}}\;{\text{glucan}}\;{\text{in}}\;{\text{residue}}}}} \right. \kern-0pt} {{\text{Amount}}\;{\text{of}}\;{\text{glucan}}\;{\text{in}}\;{\text{residue}}}} $$

7 $$ {\text{Xylose}}\;{\text{enzymatic}}\;{\text{hydrolysis}}\;{\text{yield}}\;\left( \% \right) = 100 \times {{\left( {0. 8 8\times {\text{Amount}}\;{\text{of}}\;{\text{released}}\;{\text{xylose}}\;{\text{after}}\;{\text{enzymatic}}\;{\text{hydrolysis}}} \right)} \mathord{\left/ {\vphantom {{\left( {0. 8 8\times {\text{Amount}}\;{\text{of}}\;{\text{released}}\;{\text{xylose}}\;{\text{after}}\;{\text{enzymatic}}\;{\text{hydrolysis}}} \right)} {{\text{Amount}}\;{\text{of}}\;{\text{xylan}}\;{\text{in}}\;{\text{residue}}}}} \right. \kern-0pt} {{\text{Amount}}\;{\text{of}}\;{\text{xylan}}\;{\text{in}}\;{\text{residue}}}} $$

8 $$ {\text{Total}}\;{\text{glucose}}\;{\text{yield}}\;\left( {{\text{Total}}_{{{\text{Glu}}.}} ,\;\% } \right) = 100 \times {{\left( {0. 9\times {\text{Amount}}\;{\text{of}}\;{\text{glucose}}\;{\text{in}}\;{\text{residue}} \times {\text{glucose hydrolysis yield}}} \right)} \mathord{\left/ {\vphantom {{\left( {0. 9\times {\text{Amount}}\;{\text{of}}\;{\text{glucose}}\;{\text{in}}\;{\text{residue}} \times {\text{glucose hydrolysis yield}}} \right)} {{\text{Amount}}\;{\text{of}}\;{\text{glucan}}\;{\text{in}}\;{\text{raw}}\;{\text{material}}}}} \right. \kern-0pt} {{\text{Amount}}\;{\text{of}}\;{\text{glucan}}\;{\text{in}}\;{\text{raw}}\;{\text{material}}}} $$

9 $$ {\text{Total}}\;{\text{xylose}}\;{\text{yield}}\;\left( {{\text{Total}}_{{{\text{Xyl}}.}} ,\;\% } \right) = 100 \times {{\left( {0. 8 8\times {\text{Amount}}\;{\text{of}}\;{\text{xylose}}\;{\text{in}}\;{\text{residue}} \times {\text{xylan}}\;{\text{hydrolysis}}\;{\text{yield}}} \right)} \mathord{\left/ {\vphantom {{\left( {0. 8 8\times {\text{Amount}}\;{\text{of}}\;{\text{xylose}}\;{\text{in}}\;{\text{residue}} \times {\text{xylan}}\;{\text{hydrolysis}}\;{\text{yield}}} \right)} {{\text{Amount}}\;{\text{of}}\;{\text{xylan}}\;{\text{in}}\;{\text{raw}}\;{\text{material}}}}} \right. \kern-0pt} {{\text{Amount}}\;{\text{of}}\;{\text{xylan}}\;{\text{in}}\;{\text{raw}}\;{\text{material}}}} $$

#### Enzymatic hydrolysis of extracted xylan

The enzymatic hydrolysis of extracted xylan from the liquid fraction of NaOH–ethanol-pretreated samples was carried out in phosphate buffer with a final solid loading of 5% (w/v) and 20 BXU/g substrate of xylanase. Sodium azide (10 mM) was added to the solution to prevent microbial contamination. The enzymatic hydrolysis process was incubated at optimum pH and optimum temperature for each enzyme (EpXYN1 and EpXYN3 at pH 4.8, 50 °C; XynII at pH 7.0, 60 °C) at 150 rpm in a shaking incubator for 24 h. The XOS in the supernatant was analyzed using ion chromatography (Dionex ICS-3000, USA).

### Experimental design and statistical analysis

The various process parameters involved in NaOH–ethanol pretreatment of different sorghum stem parts were evaluated using Box–Behnken design of Design Expert 11. The variables selected were NaOH loading (0.5–2%, w/v), ethanol content (10–70%, v/v), temperature (60–70 °C) and time (1–4 h). The analytical responses were *p*CA release yield (%) and xylan recovery yield (%). A total of 29 runs were designed and 5 replicates were performed at the center point.

### Analytical methods

The contents of esterified phenolic acid (*p*CA and ferulic acid) in the sorghum pith, rind and whole stem were analyzed according to the method described by Jiang et al. [8]. The structural components of glucan, xylan, arabinan and lignin of raw and pretreated samples were determined using the two-step acid hydrolysis method according to the National Renewable Energy Laboratory standard procedure but the ethanol extractives in samples were not removed prior to the determination [45]. Glucose, xylose, arabinose, acetic acid, levulinic acid, furfural and HMF were determined using HPLC (Agilent 1100, USA) consisting of refractive index detector and a column of Bio-Rad Aminex HPX-87H column (300 mm × 7.8 mm; USA). Column temperature was 55 °C using 5 mM H_2_SO_4_ as the mobile phase with flow rate of 0.6 mL/min. All experiments were performed in triplicate and the average data were reported.

Functional group changes of the raw material and the pretreated residue were examined by FTIR spectroscopy (Bruker VERTEX 80 V, Germany). The sample was scanned in the range from 4000 to 400/cm. SEM was used to observe the microstructure of the samples before and after pretreatment. Sample photographs were taken at a magnification of 1000 using FEI Quanta 200 (USA). The crystal structure of the lignocellulosic materials was analyzed using XRD. X-ray diffractometer (Rigaku Ultima IV, Japan) uses Cu-Kα (k = 1.54 Å) as a radiation source. The scan range was 5°–50° and the scan rate was 10°/min. The CrI of the crystalline sample was calculated from the XRD peak according to the following equation:

10 $$ {\text{CrI}} = 100\% \times {{\left( {I_{002} - I_{\text{am}} } \right)} \mathord{\left/ {\vphantom {{\left( {I_{002} - I_{\text{am}} } \right)} {I_{002} }}} \right. \kern-0pt} {I_{002} }} $$

where *I*_002_ is the intensity of the crystallinity peak at 2θ ≈ 22.5°, and *I*_am_ the intensity of amorphous cellulose between lattice planes of 101 and 002 at 2θ of 18.7°.

### Overall mass balance

The overall mass balance of each process stage is described as Fig. 5. First of all, 20 g of the each sample (sorghum pith, rind or whole stem) was separately pretreated by NaOH–ethanol solution (400 mL) as described in Fig. 5. The liquid fraction was separated from the solid fraction by vacuum filtration after cooling. Subsequently, the solid fraction was washed with water until neutral pH was achieved followed by drying at 65 °C to enzymatic hydrolysis. The pH of liquid fraction was adjusted to 1–2 with HCl, and 1.5 times the volume of absolute ethanol was added to the supernatant and stored at 4 °C for 24 h. Xylan precipitate was separated by centrifugation and dried for enzymatic hydrolysis, then ethanol was recovered using a rotary evaporator. Distilled water was added to the remaining liquid to replenish the original volume, and centrifuge to remove lignin. Macroporous adsorption resins D101 was used to adsorb *p*CA in the supernatant and desorbed with 70% (v/v) ethanol. *P*CA in the eluent was detected by HPLC.

## Supplementary information

**Additional file 1.** Additional figures and tables.

## Data Availability

All data generated or analyzed during this study are included in this manuscript and its additional file.

## Abbreviations

*p*CA: *p*-Coumaric acid
FTIR: Fourier transform infrared
SEM: Scanning electron microscopy
XRD: X-ray diffraction
HMF: 5-Hydroxymethyl furfural
HPLC: High-performance liquid chromatography
FPU: Filter paper units
CBU: Cellobiase units
BXU: Birch xylan units
XOS: Xylooligosaccharides
CrI: Refractive index
