# Supplementary material for: Alkaline organosolv pretreatment of different sorghum stem parts for enhancing the total reducing sugar yields and p-coumaric acid release
Source: Biotechnol Biofuels. 2020 Jun 10;13:106. doi: 10.1186/s13068-020-01746-4 (PMC7288516; doi:10.1186/s13068-020-01746-4)
Supplement: Supplementary file 1 — Additional file 1. Additional figures and tables. [file 13068_2020_1746_MOESM1_ESM.docx]

**Additional data for article**

**Captions**

**Table S1.** ANOVA of the adjusted models of NaOH-ethanol pretreatment of sorghum pith, rind and whole stem

**Table S2.** The wavelength assignments of the lignin, cellulose and hemicellulose related bands.

**Table S3**. The yields of xylose (X1) and XOS (X2-X6) of extracted xylan of sorghum pith, rind and whole stem after enzymatic hydrolysis.

**Fig. S1.** The linear correlation between the released *p*-coumaric acid and the recovered xylan. (A) Pith, (B) Rind, and (C) Whole stem.

**Fig. S2.** FTIR spectra of raw and pretreated sorghum samples. (A) Pith, (B) Rind, and (C) Whole stem.

**Fig. S3.** SEM images of raw and pretreated sorghum samples. (A) Raw pith, (D) Raw rind, (G) Raw whole stem, (B) NaOH-ethanol pretreated pith, (E) NaOH-ethanol pretreated rind, (H) NaOH-ethanol pretreated whole stem, (C) NaOH pretreated pith, (F) NaOH pretreated rind, and (I) NaOH pretreated whole stem.

**Fig. S4.** XRD patterns of raw and pretreated sorghum samples. (A) Pith, (B) Rind, and (C) Whole stem.

**Table S1.** ANOVA of the models.

Pith:

| Source | Sum of Squares | DF | Mean Square | *F-*value | *P*-value |  |
| --- | --- | --- | --- | --- | --- | --- |
| *P-coumaric acid* |  |  |  |  |  |  |
| Model | 12245.85 | 14 | 874.70 | 52.42 | < 0.0001 | significant |
| A-NaOH | 9049.72 | 1 | 9049.72 | 542.37 | < 0.0001 |  |
| B-Ethanol | 589.68 | 1 | 589.68 | 35.34 | < 0.0001 |  |
| C-Tempertature | 268.47 | 1 | 268.47 | 16.09 | 0.0013 |  |
| D-Time | 339.63 | 1 | 339.63 | 20.35 | 0.0005 |  |
| AB | 10.60 | 1 | 10.60 | 0.6350 | 0.4388 |  |
| AC | 72.93 | 1 | 72.93 | 4.37 | 0.0553 |  |
| AD | 29.43 | 1 | 29.43 | 1.76 | 0.2054 |  |
| BC | 11.53 | 1 | 11.53 | 0.6908 | 0.4198 |  |
| BD | 244.30 | 1 | 244.30 | 14.64 | 0.0019 |  |
| CD | 5.13 | 1 | 5.13 | 0.3075 | 0.5880 |  |
| A² | 1596.78 | 1 | 1596.78 | 95.70 | < 0.0001 |  |
| B² | 57.60 | 1 | 57.60 | 3.45 | 0.0843 |  |
| C² | 58.47 | 1 | 58.47 | 3.50 | 0.0823 |  |
| D² | 6.39 | 1 | 6.39 | 0.3828 | 0.5460 |  |
| Residual | 233.60 | 14 | 16.69 |  |  |  |
| Lack of Fit | 228.68 | 10 | 22.87 | 18.59 | 0.0063 | significant |
| Pure Error | 4.92 | 4 | 1.23 |  |  |  |
| R^2^ | 0.9813 |  |  |  |  |  |
| Adjusted R^2^ | 0.9626 |  |  |  |  |  |
| Predicted R^2^ | 0.8938 |  |  |  |  |  |
|  |  |  |  |  |  |  |
| *Hemicellulose* |  |  |  |  |  |  |
| Model | 1566.63 | 14 | 111.90 | 17.76 | < 0.0001 | significant |
| A-NaOH | 1170.98 | 1 | 1170.98 | 185.80 | < 0.0001 |  |
| B-Ethanol | 127.86 | 1 | 127.86 | 20.29 | 0.0005 |  |
| C-Tempertature | 1.47 | 1 | 1.47 | 0.2332 | 0.6366 |  |
| D-Time | 2.31 | 1 | 2.31 | 0.3672 | 0.5542 |  |
| AB | 1.17 | 1 | 1.17 | 0.1851 | 0.6736 |  |
| AC | 4.93 | 1 | 4.93 | 0.7820 | 0.3915 |  |
| AD | 3.61 | 1 | 3.61 | 0.5728 | 0.4617 |  |
| BC | 32.09 | 1 | 32.09 | 5.09 | 0.0405 |  |
| BD | 93.51 | 1 | 93.51 | 14.84 | 0.0018 |  |
| CD | 24.65 | 1 | 24.65 | 3.91 | 0.0680 |  |
| A² | 63.71 | 1 | 63.71 | 10.11 | 0.0067 |  |
| B² | 9.82 | 1 | 9.82 | 1.56 | 0.2325 |  |
| C² | 40.91 | 1 | 40.91 | 6.49 | 0.0232 |  |
| D² | 37.21 | 1 | 37.21 | 5.90 | 0.0291 |  |
| Residual | 88.23 | 14 | 6.30 |  |  |  |
| Lack of Fit | 87.13 | 10 | 8.71 | 31.61 | 0.0023 | significant |
| Pure Error | 1.10 | 4 | 0.2757 |  |  |  |
| Cor Total | 1654.87 | 28 |  |  |  |  |
| R^2^ | 0.9467 |  |  |  |  |  |
| Adjusted R^2^ | 0.8934 |  |  |  |  |  |
| Predicted R^2^ | 0.6957 |  |  |  |  |  |

Rind:

| Source | Sum of Squares | DF | Mean Square | *F*-value | *P*-value |  |
| --- | --- | --- | --- | --- | --- | --- |
| *P-coumaric acid* |  |  |  |  |  |  |
| Model | 18566.46 | 14 | 1326.18 | 39.44 | < 0.0001 | significant |
| A-NaOH | 13563.65 | 1 | 13563.65 | 403.37 | < 0.0001 |  |
| B-Ethanol | 513.26 | 1 | 513.26 | 15.26 | 0.0016 |  |
| C-Temperature | 152.65 | 1 | 152.65 | 4.54 | 0.0513 |  |
| D-Time | 654.16 | 1 | 654.16 | 19.45 | 0.0006 |  |
| AB | 101.30 | 1 | 101.30 | 3.01 | 0.1046 |  |
| AC | 1.30 | 1 | 1.30 | 0.0386 | 0.8470 |  |
| AD | 6.43 | 1 | 6.43 | 0.1911 | 0.6687 |  |
| BC | 1.39 | 1 | 1.39 | 0.0414 | 0.8417 |  |
| BD | 251.06 | 1 | 251.06 | 7.47 | 0.0162 |  |
| CD | 13.40 | 1 | 13.40 | 0.3984 | 0.5381 |  |
| A² | 3277.73 | 1 | 3277.73 | 97.48 | < 0.0001 |  |
| B² | 171.32 | 1 | 171.32 | 5.09 | 0.0405 |  |
| C² | 115.61 | 1 | 115.61 | 3.44 | 0.0849 |  |
| D² | 40.52 | 1 | 40.52 | 1.20 | 0.2909 |  |
| Residual | 470.76 | 14 | 33.63 |  |  |  |
| Lack of Fit | 464.43 | 10 | 46.44 | 29.33 | 0.0026 | significant |
| Pure Error | 6.33 | 4 | 1.58 |  |  |  |
| Cor Total | 19037.22 | 28 |  |  |  |  |
| R^2^ | 0.9753 |  |  |  |  |  |
| Adjusted R^2^ | 0.9505 |  |  |  |  |  |
| Predicted R^2^ | 0.8590 |  |  |  |  |  |
|  |  |  |  |  |  |  |
| *Hemicellulose* |  |  |  |  |  |  |
| Model | 464.23 | 10 | 46.42 | 12.43 | < 0.0001 | significant |
| A-NaOH | 147.63 | 1 | 147.63 | 39.53 | < 0.0001 |  |
| B-Ethanol | 225.33 | 1 | 225.33 | 60.33 | < 0.0001 |  |
| C-Temperature | 3.55 | 1 | 3.55 | 0.9514 | 0.3423 |  |
| D-Time | 29.27 | 1 | 29.27 | 7.84 | 0.0119 |  |
| AB | 35.88 | 1 | 35.88 | 9.61 | 0.0062 |  |
| AC | 3.40 | 1 | 3.40 | 0.9115 | 0.3524 |  |
| AD | 0.0196 | 1 | 0.0196 | 0.0052 | 0.9430 |  |
| BC | 3.57 | 1 | 3.57 | 0.9565 | 0.3410 |  |
| BD | 14.90 | 1 | 14.90 | 3.99 | 0.0611 |  |
| CD | 0.6724 | 1 | 0.6724 | 0.1800 | 0.6764 |  |
| Residual | 67.23 | 18 | 3.73 |  |  |  |
| Lack of Fit | 66.45 | 14 | 4.75 | 24.54 | 0.0035 | significant |
| Pure Error | 0.7737 | 4 | 0.1934 |  |  |  |
| Cor Total | 531.46 | 28 |  |  |  |  |
| R^2^ | 0.8735 |  |  |  |  |  |
| Adjusted R^2^ | 0.8032 |  |  |  |  |  |
| Predicted R^2^ | 0.6212 |  |  |  |  |  |

Whole stem:

| Source | Sum of Squares | DF | Mean Square | *F*-value | *P*-value |  |
| --- | --- | --- | --- | --- | --- | --- |
| *P-coumaric acid* |  |  |  |  |  |  |
| Model | 13421.69 | 14 | 958.69 | 39.35 | < 0.0001 | significant |
| A-NaOH | 10038.13 | 1 | 10038.13 | 412.01 | < 0.0001 |  |
| B-Ethanol | 534.40 | 1 | 534.40 | 21.93 | 0.0004 |  |
| C-Temperature | 275.33 | 1 | 275.33 | 11.30 | 0.0047 |  |
| D-Time | 355.67 | 1 | 355.67 | 14.60 | 0.0019 |  |
| AB | 34.28 | 1 | 34.28 | 1.41 | 0.2553 |  |
| AC | 75.26 | 1 | 75.26 | 3.09 | 0.1007 |  |
| AD | 5.00 | 1 | 5.00 | 0.2050 | 0.6576 |  |
| BC | 0.9506 | 1 | 0.9506 | 0.0390 | 0.8463 |  |
| BD | 222.01 | 1 | 222.01 | 9.11 | 0.0092 |  |
| CD | 3.46 | 1 | 3.46 | 0.1420 | 0.7120 |  |
| A² | 1847.33 | 1 | 1847.33 | 75.82 | < 0.0001 |  |
| B² | 77.75 | 1 | 77.75 | 3.19 | 0.0957 |  |
| C² | 38.85 | 1 | 38.85 | 1.59 | 0.2273 |  |
| D² | 9.95 | 1 | 9.95 | 0.4083 | 0.5331 |  |
| Residual | 341.09 | 14 | 24.36 |  |  |  |
| Lack of Fit | 335.26 | 10 | 33.53 | 23.01 | 0.0042 | significant |
| Pure Error | 5.83 | 4 | 1.46 |  |  |  |
| Cor Total | 13762.78 | 28 |  |  |  |  |
| R^2^ | 0.9752 |  |  |  |  |  |
| Adjusted R^2^ | 0.9504 |  |  |  |  |  |
| Predicted R^2^ | 0.8590 |  |  |  |  |  |
|  |  |  |  |  |  |  |
| *Hemicellulose* |  |  |  |  |  |  |
| Model | 1097.38 | 14 | 78.38 | 19.23 | < 0.0001 | significant |
| A-NaOH | 626.41 | 1 | 626.41 | 153.69 | < 0.0001 |  |
| B-Ethanol | 218.79 | 1 | 218.79 | 53.68 | < 0.0001 |  |
| C-Temperature | 0.3502 | 1 | 0.3502 | 0.0859 | 0.7737 |  |
| D-Time | 3.82 | 1 | 3.82 | 0.9371 | 0.3494 |  |
| AB | 15.44 | 1 | 15.44 | 3.79 | 0.0719 |  |
| AC | 10.82 | 1 | 10.82 | 2.66 | 0.1255 |  |
| AD | 0.4096 | 1 | 0.4096 | 0.1005 | 0.7559 |  |
| BC | 18.23 | 1 | 18.23 | 4.47 | 0.0528 |  |
| BD | 11.22 | 1 | 11.22 | 2.75 | 0.1193 |  |
| CD | 11.46 | 1 | 11.46 | 2.81 | 0.1158 |  |
| A² | 167.27 | 1 | 167.27 | 41.04 | < 0.0001 |  |
| B² | 9.27 | 1 | 9.27 | 2.27 | 0.1537 |  |
| C² | 0.6986 | 1 | 0.6986 | 0.1714 | 0.6851 |  |
| D² | 1.72 | 1 | 1.72 | 0.4210 | 0.5269 |  |
| Residual | 57.06 | 14 | 4.08 |  |  |  |
| Lack of Fit | 56.14 | 10 | 5.61 | 24.48 | 0.0037 | significant |
| Pure Error | 0.9175 | 4 | 0.2294 |  |  |  |
| Cor Total | 1154.44 | 28 |  |  |  |  |
| R^2^ | 0.9506 |  |  |  |  |  |
| Adjusted R^2^ | 0.9011 |  |  |  |  |  |
| Predicted R^2^ | 0.7186 |  |  |  |  |  |

**Table S2.** The wavelength assignments of the lignin, cellulose and hemicellulose related bands.

| Wavenumber (cm^-1^) | Wavelength Assignment |
| --- | --- |
| 3432 | O_2_-H_2。。。_O_6_ intramolecular stretching modes in cellulose |
| 1732 | C=O vibration of esters, ketones, aldehydes in hemicellulose and/or lignin |
| 1513 | Aromatic skeletal ring in lignin |
| 1399 | C-H deformation in cellulose and hemicellulose |
| 1254 | C-O stretch in lignin and C-O linkage in guaiacyl aromatic groups |
| 1162 | C-O-C vibrations in cellulose and hemicellulose |
| 1106 | Aromatic skeletal and C-O stretch in lignin |
| 1048 | C-O stretch in cellulose and hemicellulose |
| 896 | C-H deformation in cellulose |

**Table S3**. The yields of xylose (X1) and XOS (X2-X6) after enzymatic hydrolysis of extracted xylan of sorghum pith, rind and whole stem.

| Source | Xylanase | X1 (%) | XOS (%) | | | | |
| --- | --- | --- | --- | --- | --- | --- | --- |
|  |  |  | X2 | X3 | X4 | X5 | X6 |
| Pith | EpXYN1 | 3.70 | 15.92 | 9.93 | 4.44 | 0.54 | 0.36 |
|  | EpXYN3 | 2.74 | 10.00 | 7.12 | 0.72 | 0.77 | 0.23 |
|  | XynII | 0.84 | 6.87 | 6.71 | n.d.^a^ | 0.10 | 0.09 |
| Rind | EpXYN1 | 3.20 | 15.33 | 10.67 | 6.32 | 0.27 | 0.12 |
|  | EpXYN3 | 2.11 | 5.92 | 8.56 | n.d. | 0.86 | n.d. |
|  | XynII | 0.55 | 6.24 | 6.24 | n.d. | n.d. | 0.50 |
| Whole stem | EpXYN1 | 3.03 | 14.91 | 9.11 | 5.32 | 0.22 | 0.72 |
|  | EpXYN3 | 4.31 | 11.79 | 4.84 | 0.44 | 0.17 | 0.11 |
|  | XynII | 0.82 | 8.02 | 7.94 | n.d. | n.d. | n.d. |

^a^ n.d., not detected.


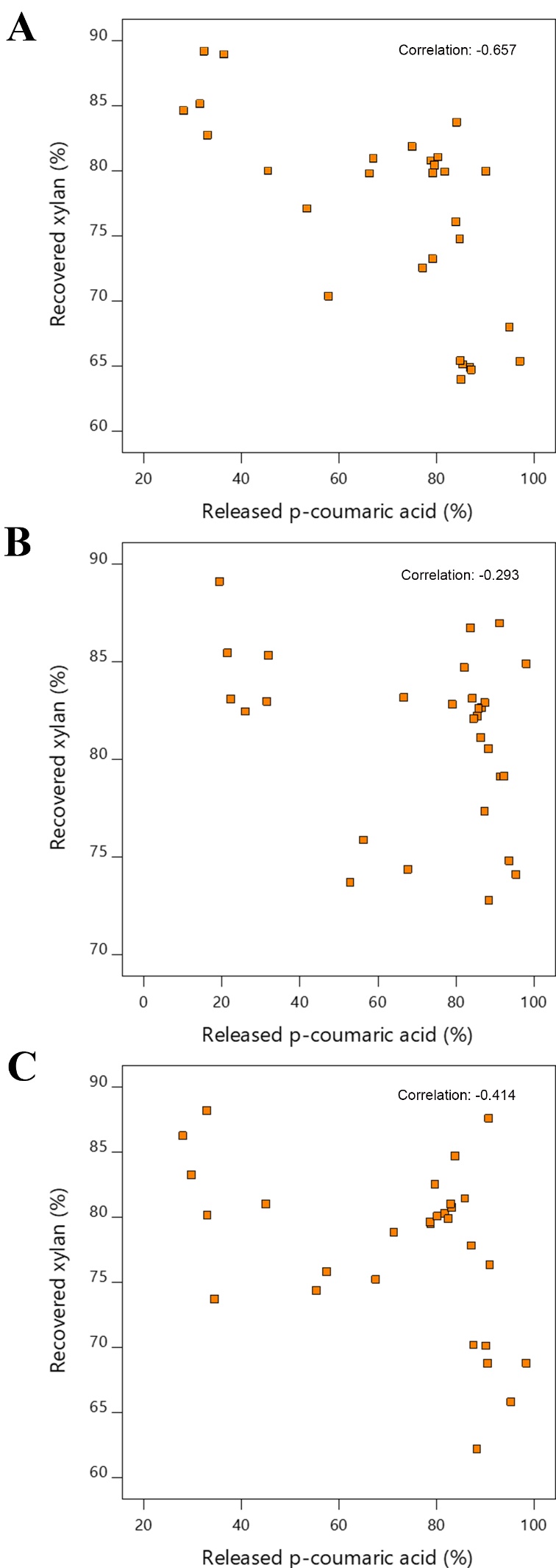


Figure S1


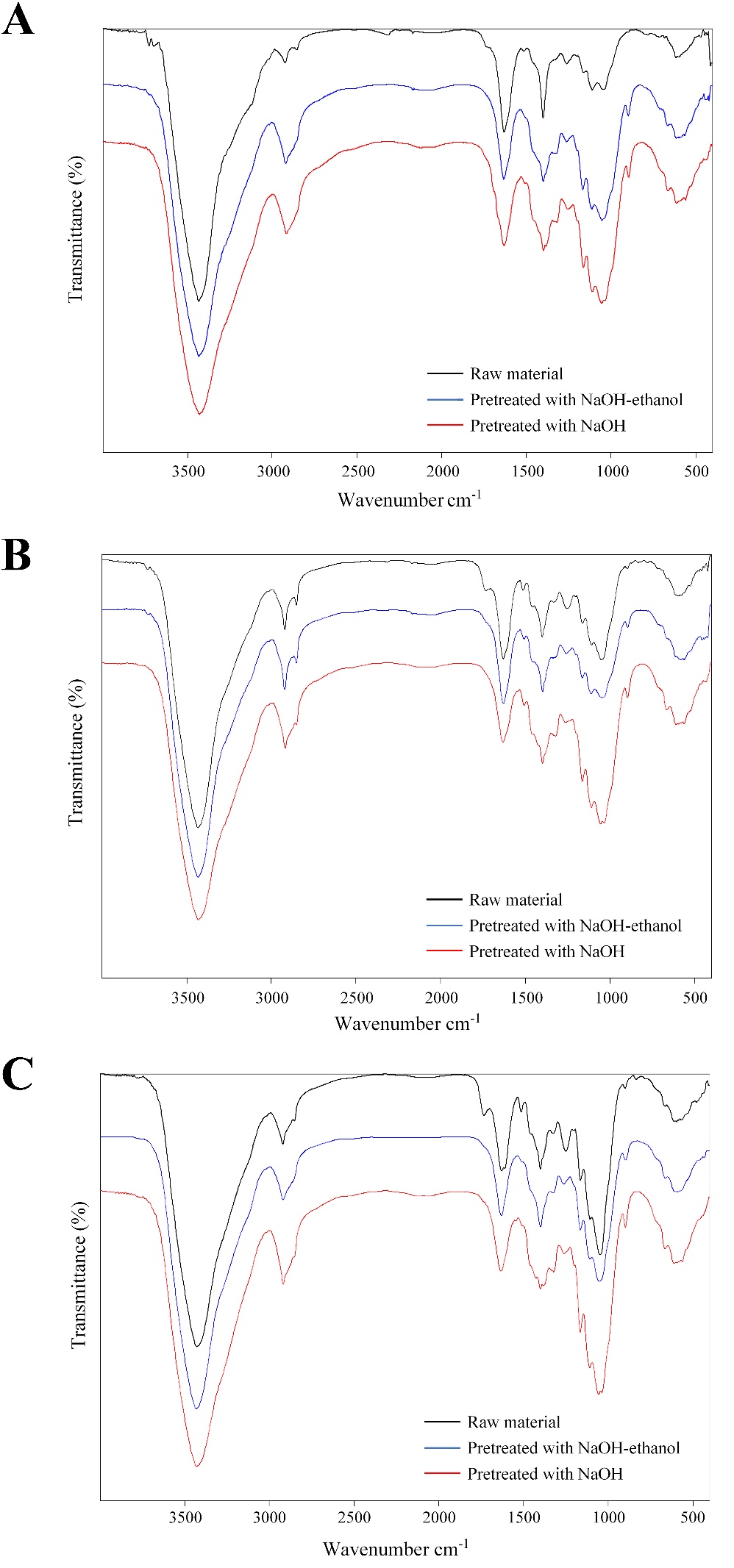
 Figure S2


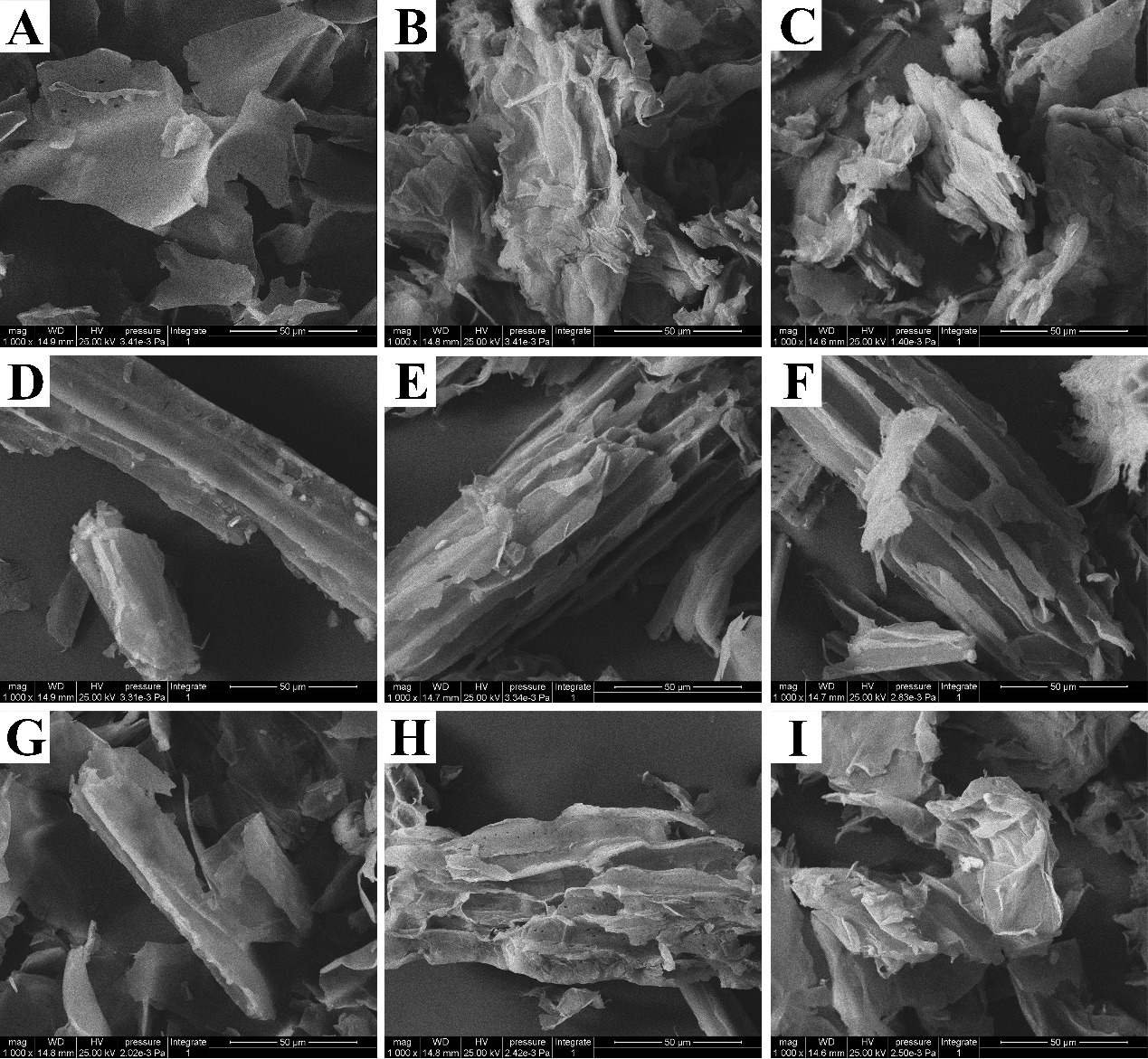


Figure S3


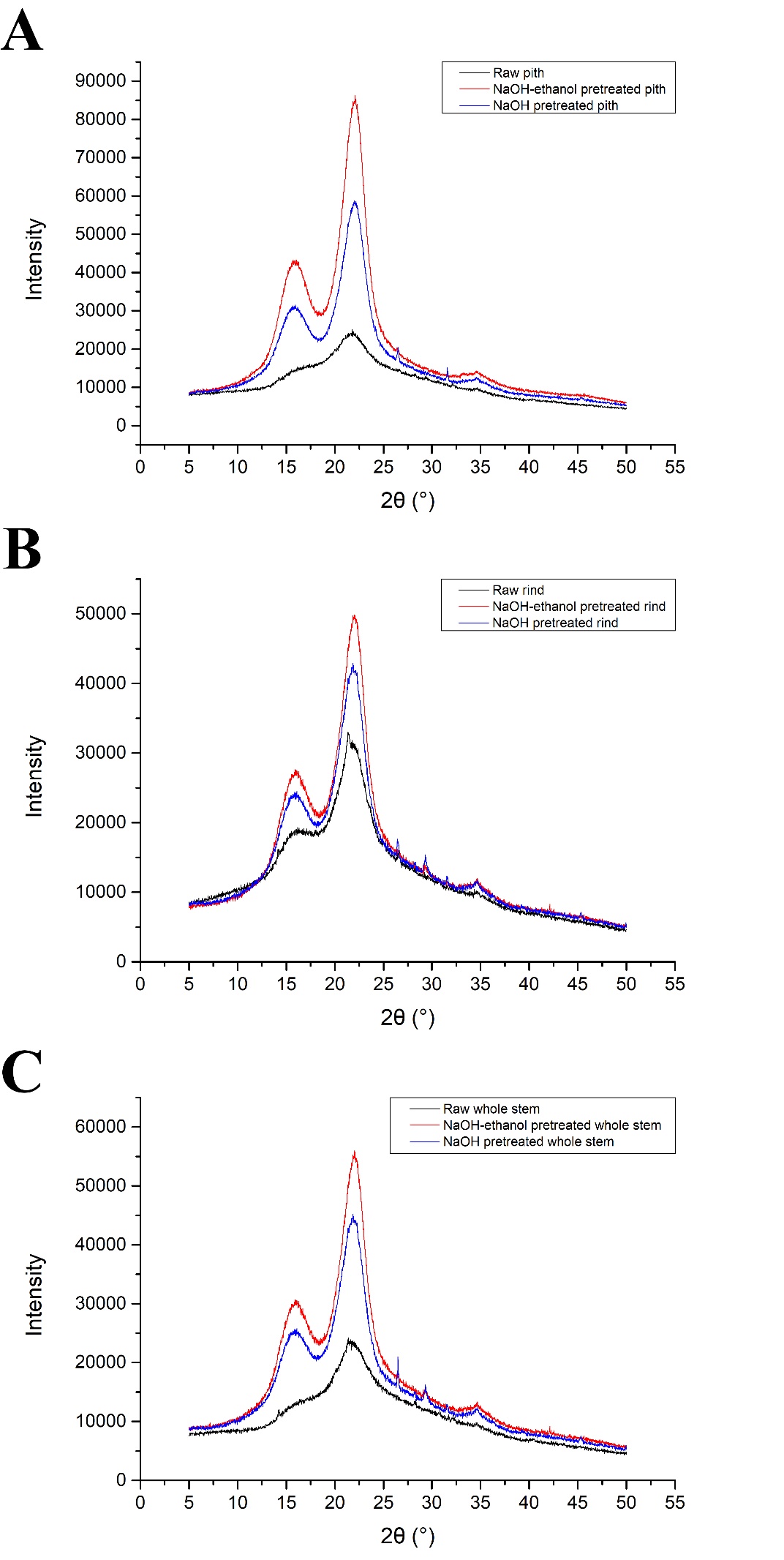
 Fig. S4
